# Physical Activity, Body Image, and Emotional Intelligence Differences in Adults with Overweight and Obesity

**DOI:** 10.3390/diseases11020071

**Published:** 2023-05-10

**Authors:** Marilyn Gilyana, Alexios Batrakoulis, Vasiliki Zisi

**Affiliations:** Department of Physical Education and Sport Science, University of Thessaly, 42100 Trikala, Greece; abatrakoulis@uth.gr (A.B.); vzisi@pe.uth.gr (V.Z.)

**Keywords:** physical activity, emotional intelligence, body mass index, body image, pedometer

## Abstract

Physical activity (PA) and emotional intelligence (EI) are integral parts of human nature. Body image (BI) and body mass index (BMI) may be indications of the psycho-emotional and physical health of human beings. The purpose of this study was to investigate the relationship between PA and EI of Greek adults living with overweight and obesity, as well as to identify the BI and EI differences in this population. A cross-sectional study design was used, involving 216 participants (65% females) of whom 51.4% were young adults (20–40 years), 48.6% were middle-aged adults (41–60 years), while 51.4% of participants were living with overweight or obesity. According to the results, all indicators of PA had very low correlations with EI factors, while statistically significant correlations were observed only for PA at work and the total score of the International Physical Activity Questionnaire with the use of emotions (*r* = 0.16 and *r* = 0.17, respectively, *p* < 0.05). Women had significantly higher EI scores than men regarding the care and empathy factor, while the individuals with obesity had lower scores in the use of emotions factor. Regarding BI, young adults who were satisfied with their BI had better control of feelings than the middle-aged adult counterparts. In conclusion, BI satisfaction and EI may differ between individuals living with overweight and obesity in both genders. Younger individuals with obesity may compensate better for their BI and better control their emotions. On the other side, PA does not seem to have an important role in these associations.

## 1. Introduction

Physical activity (PA) and emotions are important elements of human nature. According to the World Health Organization, PA is defined as “any form of muscular effort that increases energy expenditure above resting levels, and is categorized in terms of type, intensity and purpose”, while exercise is a subset of PA that is planned, structured, and repetitive, aiming to improve or maintain various physical fitness parameters [1]. According to the current guidelines on PA and sedentary behavior, regular PA and exercise should be a foundational part of a healthy lifestyle for the masses, including those who are living with overweight or obesity [2]. Importantly, exercise for weight loss has been recently reported as one of the most popular trends in the health and fitness industry at global [3], regional [4], and national levels [5,6]. Emotional intelligence (EI) is also a topic that has gained significant recognition from scientists, psychologists, educators, and health scholars over recent decades. The term “emotional intelligence” was first formulated by Mayer and Salovey [7] and they clarified that it is social intelligence and includes: (i) the ability to recognize and distinguish one’s own emotions and those of others; (ii) distinguishing good feelings from bad and using the good to transform the bad; and (iii) using this information to guide one’s thoughts and actions.

EI is concerned with understanding the self and others as well as adapting to successfully cope with environmental demands [8]. EI can also affect logical thinking as it is correlated negatively with logical thinking and stress, while logical thinking weakens with age, as recently reported [9]. PA and EI are correlated positively in all dimensions. Engaging in physical activity can affect all EI dimensions positively. Interestingly, high levels of EI can decrease violent behavior among university students [10]. Body image (BI) is the mental representation that each person has of their body; it can be positive and associated with positive emotions (e.g., self-respect, self-esteem, well-being, and optimism) or a negative one associated with negative emotions accordingly [11]. Only a few review studies have investigated the relationship between BI and obesity, showing weak evidence.

According to the current evidence, there are two concepts regarding the association between BI and obesity. On the one side, changes in BI are accompanied by weight loss, but on the other side, such changes are possible using psychotherapy independent of weight loss. Relevant studies show that BI dissatisfaction increases with increasing weight, which is more pronounced in women than men [12,13]. In contrast, some other countries prefer large body sizes, underestimating their weight as well as obesity-related risks due to cultural factors [14]. A narrative review by McNabney [15] showed that BI dissatisfaction can independently affect the sexual response cycle and mental health results, but in those cases where excess weight supports higher levels of BI satisfaction, it appears to be contributing to their sexual satisfaction too. In a study that examined the interaction between EI and BI, it was shown that a positive view of others had a greater effect on self-esteem in men than in women, and a positive self-view in women had a greater effect on the ability to repair emotions than in men [16].

To date, there are no relevant studies providing data regarding the relationship between PA and EI among individuals with unhealthy weight. Therefore, this investigation aimed to investigate the relationship between PA and EI of Greek adults living with overweight and obesity, as well as to identify the BI and EI differences in this population. We hypothesized that (i) greater PA levels would elicit higher EI responses among individuals with overweight or obesity and (ii) no BI and EI differences would be observed between the age groups and genders. The outcomes of the present study will support clinicians and practitioners to promote the vital role of regular PA in health behavior among people living with excess weight.

## 2. Materials and Methods

### 2.1. Participants

A total of 216 individuals (76 men and 140 women) aged 20–60 years were randomly selected from different regions of Greece (Central Greece, Thessaly, and Epirus) to participate in this study. Participants were divided into the following two age groups: (i) 20–40 years old (*n* = 111) and (ii) 41–60 years old (*n* = 105). In brief, they had a mean age of 39.7 years ± 20.3 years. In the sample, all body mass index (BMI) classifications were included (overweight: 5%, normal weight: 44%, overweight: 28%, obesity: 23%). The educational profile of the sample was quite high with 63% being graduates of higher education and holders of postgraduate degrees, 29% being graduates of secondary education, and only 5% being graduates of primary education. Permission was requested from the Outpatient Clinics for Disorders of Lipid Metabolism and Obesity of the University General Hospital of Ioannina to allow access to people with overweight and obesity who visit these clinics to regulate their weight. In addition, people from various gyms who demonstrated sufficient physical activity levels participated in the study. All participants were fully informed of the research and the participation process. A condition for participation was that the subjects were healthy and were not on medication or under medical monitoring. They signed a consent letter and completed the questionnaires required. About half of the participants (*n* = 106) were asked to wear a pedometer device for four consecutive days so that their total steps were recorded, and their physical activity was assessed and compared with the physical activity questionnaire. Of the four days, two days were required to be a weekend or holiday. Table 1 shows the distribution of the sample by BMI and age group.

### 2.2. Questionnaires and Measuring Instruments

The Greek EI scale [17] was used, which was based on the approaches of Mayer and Salovey [7] and was designed exclusively to measure EI in the Greek population. This specific scale consists of 52 questions and includes four factors: (a) expression and recognition of emotions, (b) control of emotions, (c) use of emotions, and (d) empathy and care. From early tests, this scale appeared to have a validity and reliability coefficient value of 0.85 and 0.89, respectively [17].

The 9-point BI scale [18] was used, which has been used in the Greek population [19]. According to this scale, each examinee is asked to choose which of the nine body sketches they believe represents (a) the body they have now, (b) the body they would like to have, and (c) the ideal body. For the statistical analysis, a discrete variable with three levels was used: (a) satisfied, where the body they thought they had was similar to the body they would like to have, (b) low satisfaction, where the body they thought they had was only one unit away from their desired body, and (c) dissatisfied with their body, where the body they thought they had was 2 units or more away from their desired body. According to the test–retest reliability test, the intra-class correlation coefficient was 0.9 [18].

Given that PA is defined as any skeletal muscle-driven movement of the body that necessitates energy consumption, all movement, whether carried out for recreation, transportation to get to and from locations, or as part of a person’s job, is considered PA [1]. PA was assessed using the long form of the international PA questionnaire (IPAQ) for the last seven days. The IPAQ is a 27-item self-reported measure of PA for use with adults aged 15 to 69 years old [20] and includes five parts. The first four parts record the frequency (hours/day and days/week) and intensity of PA (intense and moderate intensity) related to (a) work, (b) commuting, (c) housework and family care, and (d) leisure and exercise. The fifth and last part records the time spent on sedentary activities. This questionnaire was translated into Greek by Makavellou et al. [21] and was checked by a group of bilingual individuals with repeated measurements over a period of one week in 20 males and 20 females aged 20–45 years with a reliability coefficient value of 0.82, while the corresponding value mentioned in the international literature is 0.80.

Participants were measured for height and weight with valid measurement tools available at weight management clinics or gyms. BMI is defined as the quotient of body weight in kilograms divided by height in meters squared. Values below 18.5 are considered underweight. An adult with a BMI of 18.5–24.9 kg/m^2^ is considered an individual with normal weight, 25–29.9 kg/m^2^ with overweight, and 30 kg/m^2^ with obesity [22].

An easy-to-use pedometer (YAMAX Digi-Walker SW-200) was used to record the steps of 106 participants who wore it for four consecutive days, including two working days and two holidays. The pedometer was worn continuously except for sleeping, resting, and bathing hours and the recording was continuous. Indications for the number of steps are as follows: <5000 steps per day are considered low PA, characterizing sedentary people; 5000–7499 steps/day are considered low activity; 7500–9999 steps/day are considered somewhat good activity; ≥10,000 are considered very good activity; and >12,500 are characterized as high activity [23].

### 2.3. Statistical Analyses

Two two-way multivariate analyses of variance (two-way MANOVA) were performed to examine differences in the EI factors in terms of BMI with its four categories and age group, as well as in terms of BI of three levels of satisfaction and age group. The dependent variables were the four parameters of EI: (a) use of emotions, (b) control of emotions, (c) empathy and interest, and (d) recognition and expression of emotions. For the first two-way analysis, the independent variables were the two age groups (20–40 years, 41–60 years) and BMI, of which the categories were (a) underweight, (b) normal weight, (c) overweight, and d) obesity, although we excluded the underweight group due to the lack of males in this group. For the second two-way analysis, the independent variables were age groups and BI in the three levels of (a) satisfied, (b) low satisfaction, and (c) not satisfied. To test the age groups’ interaction, separate one-way ANOVAs were performed on each age group. Pearson correlation analysis was performed between PA (a) at work, (b) commuting, (c) during housework, and (d) in free time, with the number of steps and the four parameters of EI. Statistical significance was set at *p* < 0.05. Data were analyzed using the SPSS 26.0 software (IBM Corp., Armonk, NY, USA). Results are presented as mean ± standard deviation (SD).

## 3. Results

### 3.1. Emotional Intelligence and Body Mass Index

**Figure 1:**
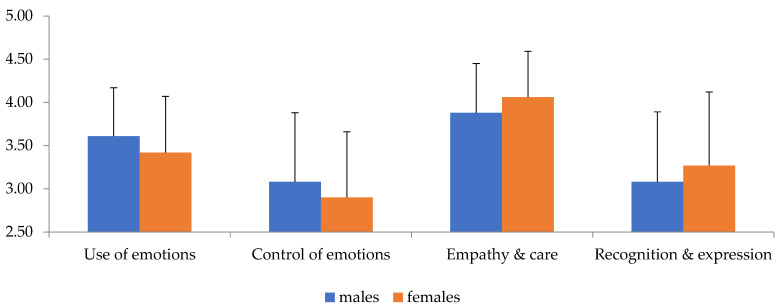
Interaction level of emotional intelligence factors (*X*-axis) with gender (*Y*-axis).

**Figure 2:**
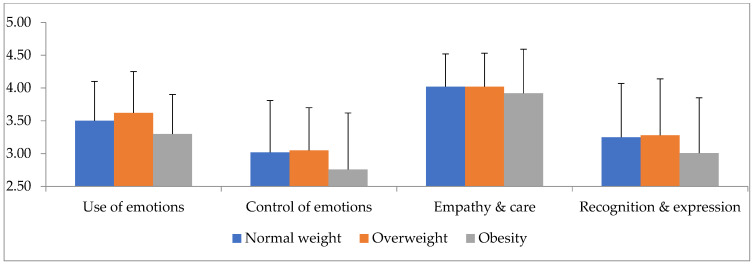
Interaction level of emotional intelligence factors (*X*-axis) with body mass index (*Y*-axis).

A statistically significant main effect of gender (Roy’s largest root = 0.101 F_4,195_ = 4.94, *p* = 0.001, *η*^2^ = 0.92) and BMI (Roy’s largest root = 0.05, F_4,196_ = 2.44, *p* = 0.049, *η*^2^ = 0.47) was found. Examining the univariate tests showed a statistically significant effect of gender for the empathy and care factor (F_1,204_ = 6.68, *p* = 0.01, *η*^2^ = 0.33), as shown in Table 2 and Figure 1, while showing how females scored better than males on this factor. A significant effect of BMI on the use of emotions was also found (F_2,204_ = 3.42, *p* = 0.035, *η*^2^ = 0.33) (Figure 2). Bonferroni’s post hoc tests found that the score of individuals with overweight, who had the highest score among the three groups, differed significantly from the score of those with obesity, who had the lowest score among the groups (MD = 0.32, *p* = 0.019).

### 3.2. Emotional Intelligence and Body Image

**Figure 3:**
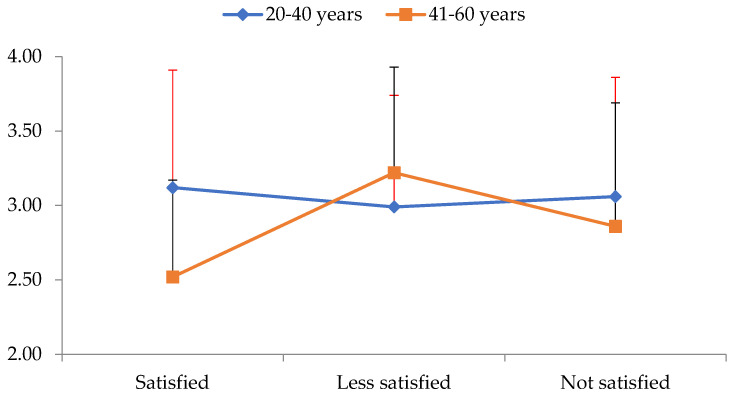
Interaction level of body image satisfaction (*X*-axis) with age group category on the emotion control factor of emotional intelligence (*Y*-axis).

Table 3 shows the mean and SD across all EI variables for all three levels of BI satisfaction. The results showed a statistically significant main effect for age (Roy’s largest root = 0.80, F_4,205_ = 4.08, *p* = 0.003, *η*^2^ = 0.74) and a statistically significant interaction level of BI satisfaction by age category (Roy’s largest root = 0.61, F_4,206_ = 3.12, *p* = 0.016, *η*^2^ = 0.57). The univariate tests showed a statistically significant effect of age on the recognition and expression of emotions factor (F_1,214_ = 13.27, *p* < 0.001, *η*^2^ = 0.60). People under 41 had a better score than older people. Additionally, a significant interaction level of satisfaction with BI by age category was found in the emotion control factor (F_2,214_ = 4.09, *p* = 0.018, *η*^2^ = 0.38) (Figure 3).

To examine this interaction, simple main effects of age group on each level of BI satisfaction were tested separately using independent *t*-tests. Statistically significant age differences were found only in subjects who were satisfied with their BI (t34 = 2.46, *p* = 0.019). As can be seen in Figure 3, among the people who were satisfied with their bodies, people over 40 years of age had much lower scores in the emotion control factor of EI variables than the younger ones.

### 3.3. Correlations of Physical Activity and Emotional Intelligence

All correlations were very low, even those that were statistically significant as shown in Table 4. Steps recorded by pedometers were significantly associated with PA during commuting and leisure time and IPAQ total score (*p* < 0.001), but not with any factor of EI. Of the EI factors, statistically significant associations emerged only in the use of emotions. Specifically, the correlation of this factor with the PA score at work and the total IPAQ score was statistically significant (*p* < 0.05). With the control of emotions there was a low positive correlation with all categories of PA except leisure-time PA, which was negative (*r* = −0.04). As for empathy and care, it is negatively correlated with PA during work (*r* = −0.13) and positively correlated with PA during commuting. Expression and recognition of emotions has a low negative correlation with all categories of PA except PA in leisure time (*r* = 0.02).

## 4. Discussion

The present study examined the effects of PA and BI on EI in individuals with overweight and obesity among Greek adults, and whether EI factors differ according to gender and age. Most of the research hypotheses were confirmed and came to strengthen previous research that examined these relationships. In our research, the PA levels of the entire sample were high and there were no large differences between individuals but, nevertheless, a significant positive correlation was found between PA at work and the emotion control factor. A significant positive correlation was shown between total PA and control of emotions. This supports previous research in that there is a positive correlation between PA and EI factors [24,25,26]. In general, various types of PA and exercise induce beneficial changes in numerous psychological health-related parameters in adults with overweight and obesity [27,28,29,30,31]. However, EI is not limited to various PA types, since it can be in any form, and it can also include traditional dancing styles [32].

The present findings showed differences between males and females in terms of EI factors, with females demonstrating superior results in empathy and care. This outcome can be explained by research conducted by Tamres et al. [33], who reported that females respond more emotionally to problems and spend more time discussing them with friends and family. In general, females score better and more than men, which has been confirmed in previous research [25,26,34]. There are studies that have shown opposite results with males being more emotionally intelligent and significantly different from women in the whole of EI or in some factors [35]. This may be due to the differences in culture that exist between West and East, specifically the leading role of the male who is more independent and powerful in Eastern societies. Additionally, profession may play a role in presenting an adequate level of EI as no differences were found between male and female nurses [36].

People with obesity appear to lag behind in EI in all factors with a significant difference in the use of emotions, which is in agreement with the research review conducted by Christodoulou [37], who reported that obesity reduces a person’s cognitive and emotional skills compared to those with normal weight. Such an observation makes the obesity problem more serious and its treatment more complicated. Furthermore, excess weight also appeared to negatively affect EI according to Migues-Torres and colleagues [36].

Most research has shown that EI improves over time. The older a person is, the more education and experience they acquire, and this in turn improves EI [36,38]. Moreover, EI is positively correlated with age [34]. The present study showed that age plays an important role in EI; however, contrary to the above, young people aged 20–40 years have better EI than adults aged 41–60 years. Taking this into consideration, it appears that in some EI factors improvement is inversely proportional to increasing age. In all factors of EI, young people are ahead except for the use of emotions where the values of the two age groups are almost the same. In all other EI factors, young people perform better than older people with differences in the expression and recognition of emotions in favor of young people. The results of the present study may be due to the age group separation that was carried out (20–40 years vs. 41–60 years). As we can see, there is a large portion of young adults aged 35–45 years that are included in both groups which shows that experience and education are included in and strengthen the first age category. Furthermore, the comparison is not between the very young and the very old participants to show the difference in experience and life stage that each group is at.

The present study examined BI in relation to EI, assuming that all those who are satisfied with their BI will have high EI, but the results did not confirm this and there were no differences in any of the three categories of BI (satisfied, low satisfaction, and unsatisfied) in relation to EI factors. This may be because BI satisfaction cannot reveal the levels of EI by itself and is influenced by other factors such as gender or age. To examine this possibility, the interaction of age with BI satisfaction in EI was tested and the results showed that among all those who were satisfied with their BI, the young adults (20–40 years) had a statistically significant difference from the middle-aged adults (41–60 years) in the control of emotions factor. Interestingly, a study showed that people who have a lower BMI are more satisfied with their BI, and the more they exercise, the more satisfied they are with their BI [19]. Recently, Stagi and colleagues [39] confirmed the association between self-perceived BI and body fat measured in both genders and in different groups of age. BI has been examined in relation to EI traits and shown to be significantly associated with both ideal and estimated body weight [40]. Females show greater BI dissatisfaction than males. Likewise, females with obesity fail more than males to attain the normal weight that represents them [41,42]. However, PA improves BI independently of weight loss [43]. More specifically, sample studies show that BI dissatisfaction increases with increasing weight and this is more pronounced in females than in males [12,13], but that is not the case in regions where individuals are more satisfied with BI classified as individuals with overweight and obesity [14]. In general, BI satisfaction may play a key role in adhering to regular PA and exercise of any kind among adults living with larger bodies. This is a critical observation since populations with unhealthy weight should be committed to physical movement, aiming to improve several cardiometabolic health-related indices through various exercise types [44,45].

Considering effective ways to interpret the present outcomes in the real world, it may be suggested that training programs are implemented in various settings (e.g., workplaces, educational institutions, local social spaces, and primary healthcare providers) in the form of seminars or information leaflets. Such an approach will create a greater awareness among support clinicians and practitioners in order to disseminate the value of regular PA among the populations with excess weight, as far as the immediate practical improvement of EI is concerned. There is also an indirect way of improving EI by increasing PA, especially in the workplace, and its positive effect on employee productivity has been proven. Another practical way to improve EI is weight loss, since the implementation of weight loss programs and weight management therapies contribute to better EI performance. With weight loss, satisfaction with BI is also achieved and this results in EI improvement. The fact that obesity prevalence is increasing in all age categories worldwide requires that these investigations be intensified so that the conclusions and results can be used to provide interventions for the treatment and prevention of obesity.

## 5. Conclusions

It is concluded that PA, especially at work, has a positive effect on EI and especially on the control of emotions factor. Females, once again, seem to outperform males in EI and especially in the empathy and care factor. As for weight, no effects were found on EI of individuals with overweight, something that needs to be further examined to confirm whether or not a little to significant excess weight affects EI as it does in individuals with obesity whose weight has a negative effect on EI. In terms of age, young people aged 20–40 years appeared to have better EI than older people. BI did not appear to influence EI, but when its interaction with age on EI was examined, it was shown that among all those who were satisfied with BI, younger individuals (20–40 years) were better than their older counterparts (41–60 years) in the control of emotions factor.

## Figures and Tables

**Table 1:** Distribution of sample according to BMI and age group.

Age Group	BMI Classification	Males (*n*)	Females (*n*)
20–40 years (*n* = 111)	Underweight	0	10
	Normal weight	14	47
	Overweight	15	8
	Obesity	8	9
41–60 years (*n* = 105)	Underweight	0	0
	Normal weight	7	27
	Overweight	22	16
	Obesity	10	23
	Total	76	140

BMI: body mass index.

**Table 2:** Means and standard deviations of the body mass index and emotional intelligence categories for both genders.

Emotional Intelligence Factor	BMI Classification	Males Mean SD	Females Mean SD	Total Mean SD
Use of emotions	Normal weight	3.77 0.44	3.43 0.63	3.50 0.60
	Overweight	3.58 0.61	3.69 0.67	3.62 * 0.63
	Obesity	3.49 0.55	3.20 0.61	3.30 * 0.60
	Total	3.61 0.56	3.42 0.65	3.49 0.62
Control of emotions	Normal weight	3.15 0.88	2.98 0.77	3.02 0.79
	Overweight	3.07 0.68	3.03 0.63	3.05 0.65
	Obesity	3.01 0.97	2.62 0.77	2.76 0.86
	Total	3.08 0.80	2.90 0.76	2.97 0.78
Empathy and care	Normal weight	3.99 0.46	4.03 0.51	4.02 0.50
	Overweight	3.88 0.51	4.23 0.46	4.02 0.51
	Obesity	3.73 0.76	4.02 0.61	3.92 0.67
	Total	3.88 ** 0.57	4.06 ** 0.53	3.99 0.55
Expression and recognition	Normal weight	3.09 0.71	3.30 0.85	3.25 0.82
	Overweight	3.14 0.90	3.48 0.78	3.28 0.86
	Obesity	2.96 0.77	3.05 0.88	3.01 0.84
	Total	3.08 0.81	3.27 0.85	3.20 0.84

** *p* < 0.01, * *p* < 0.05. BMI: body mass index; SD: standard deviation.

**Table 3:** Means and standard deviations of the body image categories and emotional intelligence parameters in both age groups.

Emotional Intelligence Factor	Body Image Category	20–40 Years Mean SD	41–60 Years Mean SD	Total Mean SD
Use of emotions	Satisfied	3.72 0.65	3.63 0.64	3.68 0.60
	Low satisfaction	3.42 0.57	3.60 0.45	3.49 0.53
	Not satisfied	3.47 0.71	3.35 0.69	3.41 0.70
	Total	3.49 0.62	3.49 0.61	3.49 0.61
Control of emotions	Satisfied	3.12 0.79	2.52 0.65	2.82 0.78
	Low satisfaction	2.99 0.75	3.22 0.71	3.08 0.74
	Not satisfied	3.06 0.80	2.86 0.83	2.95 0.82
	Total	3.03 0.77	2.94 0.79	2.99 0.78
Empathy and care	Satisfied	4.12 0.38	4.01 0.53	4.06 0.46
	Low satisfaction	3.98 0.50	3.90 0.58	3.95 0.53
	Not satisfied	4.12 0.51	3.97 0.66	4.03 0.60
	Total	4.05 0.49	3.95 0.60	4.00 0.55
Expression and recognition	Satisfied	3.44 0.84	2.89 0.93	3.16 0.92
	Low satisfaction	3.50 0.80	2.96 0.91	3.28 0.88
	Not satisfied	3.36 0.70	3.09 0.82	3.21 0.78
	Total	3.44 ** 0.77	3.01 ** 0.87	3.23 0.85

** *p* < 0.01. SD: standard deviation.

**Table 4:** Pearson coefficients for the associations between physical activity and emotional intelligence parameters.

IPAQ	Steps Recorded	Use of Emotions	Control of Emotions	Empathy and Care	Expression and Recognition
Steps recorded		0.087	0.020	0.103	0.029
At work	0.088	0.159 *	0.048	−0.125	−0.014
Commuting	0.320 **	0.105	0.046	0.120	−0.034
During housework	0.183	−0.055	0.026	0.019	−0.056
Leisure time	0.300	0.132	−0.038	0.053	0.018
Total	0.254 **	0.165 *	0.038	−0.059	−0.028

** *p* < 0.01, * *p* < 0.05. IPAQ: International Physical Activity Questionnaire.

## Data Availability

The data that support the findings of this study are available from the corresponding author upon reasonable request.
